# Differentiating Persistent Sepsis From Pembrolizumab-Induced Secondary Hemophagocytic Lymphohistiocytosis and Concurrent Immune-Mediated Nephritis: A Diagnostic and Therapeutic Challenge

**DOI:** 10.7759/cureus.115797

**Published:** 2026-09-04

**Authors:** Ahmed Nisar

**Affiliations:** 1 Internal Medicine, Luton and Dunstable Hospital, Luton, GBR; 2 Nephrology, Rehman Medical Institute, Peshawar, PAK

**Keywords:** acute kidney injury, anakinra, hemophagocytic lymphohistiocytosis, hlh, immune checkpoint inhibitor, immune-related adverse event, pembrolizumab, secondary hlh

## Abstract

Hemophagocytic lymphohistiocytosis (HLH) is a rare, life-threatening hyperinflammatory syndrome that can occur as an immune-related adverse event following immune checkpoint inhibitor therapy. Early diagnosis is challenging because its clinical features frequently overlap with severe sepsis, particularly in oncology patients who have recently received marrow-suppressing cytotoxic regimens.

We report the case of a 39-year-old woman with triple-negative breast cancer who underwent neoadjuvant chemo-immunotherapy with carboplatin, paclitaxel, epirubicin, cyclophosphamide, and pembrolizumab. Two weeks after her last cycle, she presented with fever, a generalized rash, and purulent discharge from a peripherally inserted central catheter site, with blood cultures initially positive for Gram-positive bacteria. Despite prompt microbiological clearance with broad-spectrum antimicrobials, she developed persistent daily pyrexia, progressive liver function derangement, severe anemia, bilateral pleural effusions, and stage 3 acute kidney injury requiring continuous renal replacement therapy. Serial assessments revealed marked hyperferritinemia, hypertriglyceridemia, elevated aspartate aminotransferase, and an increasing HScore. Bone marrow biopsy showed treatment-related dyspoiesis without overt hemophagocytosis, which did not preclude the diagnosis of pembrolizumab-associated secondary HLH and concurrent immune-mediated nephritis. Targeted treatment with high-dose methylprednisolone, anakinra, and subsequently mycophenolate mofetil led to clinical recovery. The patient was successfully liberated from continuous renal replacement therapy; her cytopenias and kidney function improved, and her hyperinflammatory markers and HScore normalized. Anakinra was discontinued, and she was discharged on oral mycophenolate mofetil and prednisolone.

This case underscores the complexity of distinguishing checkpoint inhibitor-associated HLH from persistent infection. Serial clinical scoring, multidisciplinary input, and substituting conventional cytotoxic HLH protocols with targeted biological therapies and organ-preserving immunosuppressants are essential to resolve the hyperinflammatory cascade while preserving renal function.

## Introduction

The introduction of immune checkpoint inhibitors (ICIs) like pembrolizumab has significantly improved survival outcomes in triple-negative breast cancer (TNBC) [1,2]. However, disinhibiting the immune system can trigger severe immune-related adverse events (irAEs) [3]. Secondary hemophagocytic lymphohistiocytosis (HLH) is a life-threatening hyperinflammatory syndrome resulting from uncontrolled activation of immune effector cells, particularly T lymphocytes and macrophages, leading to excessive cytokine release and progressive tissue and organ injury [4,5]. Because its presentation (fever, cytopenias, and organ dysfunction) closely mimics sepsis, diagnosing HLH in oncology patients receiving concurrent marrow-suppressing chemotherapy is extremely difficult [5,6]. The HScore is a validated clinical scoring system that estimates the probability of secondary HLH using readily available clinical and laboratory parameters and can support diagnosis when conventional HLH criteria are difficult to establish. The presence of concurrent immune-mediated nephritis may further support an ICI-related immune dysregulatory process when it occurs alongside other immune-related toxicities. We report a complex case of pembrolizumab-induced secondary HLH presenting with concurrent immune-mediated nephritis and initial bacterial sepsis.

## Case presentation

A 39-year-old woman was diagnosed with grade 3, triple-negative invasive ductal carcinoma of the right breast, presenting as a 5 cm mass with palpable right axillary lymphadenopathy. She commenced neoadjuvant chemo-immunotherapy with carboplatin, paclitaxel, epirubicin, cyclophosphamide, and pembrolizumab.

Two weeks following her final treatment session, she presented to the emergency department with pyrexia, a generalized cutaneous rash, and purulent discharge at her left peripherally inserted central catheter (PICC) site. She was initially managed for presumed neutropenic/catheter-related sepsis with intravenous piperacillin/tazobactam and teicoplanin. Initial blood cultures grew Gram-positive bacteria, prompting escalation to intravenous meropenem due to persistent high-grade fevers.

Subsequent repeat blood cultures demonstrated complete microbiological clearance. Extensive staging workup with diagnostic cross-sectional imaging (negative computed tomography of the thorax, abdomen, and pelvis) ruled out occult infectious foci or intra-abdominal abscesses. There was no clinical or radiological evidence of splenomegaly. Comprehensive viral polymerase chain reaction (PCR) testing was negative with the exception of low-level Epstein-Barr virus (EBV) viraemia (1,330 copies/mL). Transthoracic echocardiography showed no features of infective endocarditis, and culture of the removed PICC tip yielded no growth.

Despite documented microbiological clearance and sterile cultures, the patient continued to spike daily fevers. Given the temporal association with recent pembrolizumab exposure, the persistence of fever and progressive hyperinflammation despite microbiological clearance and broad-spectrum antimicrobial therapy, and the absence of an alternative infectious focus, pembrolizumab-associated secondary HLH was considered the most likely cause of the hyperinflammatory syndrome. Her clinical course was further complicated by worsening liver function tests and severe anaemia (haemoglobin <70 g/L) requiring packed red blood cell transfusions. Bilateral pleural effusions subsequently developed, raising clinical suspicion for secondary haemophagocytic lymphohistiocytosis (HLH). Initial laboratory evaluation confirmed marked hyperferritinaemia, hypertriglyceridaemia, and elevated aspartate aminotransferase (AST) levels. Specialised assays, including soluble interleukin-2 receptor (sCD25) and natural killer (NK) cell function, were unavailable locally. The evolution of the patient's key laboratory parameters throughout admission is summarised in Table 1. Her baseline HScore was calculated at 149 (corresponding to a 16-25% probability of HLH), prompting close clinical monitoring without immediate initiation of high-dose corticosteroids [5,7].

**Table 1:** Longitudinal summary of key laboratory parameters throughout the clinical course. Values represent representative trajectory points during the clinical presentation, peak systemic hyperinflammation/organ damage, and post-treatment recovery. Dashes (—) indicate parameters not assessed at that specific time point. ALT: alanine aminotransferase; AST: aspartate aminotransferase; HLH: hemophagocytic lymphohistiocytosis; PCR: protein-to-creatinine ratio; Prob.: probability

Parameter	Baseline / Admission	Peak Severity	Resolution / Discharge	Reference Range	Units
Hemoglobin	108	62	79	120–150	g/L
Platelets	290	45	270	150–400	×109/L
Absolute Neutrophil Count	4.2	0.8	3.5	2.0–7.5	×109/L
Serum Ferritin	3200	12100	2616	15–200	ng/mL
Fibrinogen	2.4	1.1	2.8	1.5–4.0	g/L
Triglycerides	3.2	5.8	1.9	< 1.7	mmol/L
AST	65	175	23	10–35	U/L
ALT	48	142	28	10–45	U/L
Total Bilirubin	14	38	12	3–20	μmol/L
Serum Creatinine	75	560	134	45–84	μmol/L
Urine Protein to creatinine ratio	—	1232	529	< 150	mg/g
H-Score	149	227	79	—	points
Prob. of HLH	16–25%	96–98%	< 1%	—	%

Over the subsequent five days, her clinical trajectory deteriorated. With a rising HScore of 185, oral prednisolone 40 mg daily was introduced. Shortly thereafter, she developed severe acute kidney injury (AKI) with a urine protein-to-creatinine ratio (PCR) of 1,232 mg/g, indicative of high-grade glomerular or tubulointerstitial injury. Renal investigations, including antinuclear antibody (ANA), antineutrophil cytoplasmic antibody (ANCA), anti-glomerular basement membrane (GBM) antibodies, and complement levels, were unremarkable, with no serological evidence of an alternative autoimmune or glomerular process. In the context of recent pembrolizumab exposure, concurrent immune-related HLH, significant AKI, and the absence of another identified cause, pembrolizumab-associated immune-mediated nephritis was considered the most likely cause of the renal injury. Recalculation of her HScore, incorporating worsening hyperferritinaemia, hypertriglyceridaemia, and transaminitis, demonstrated a sharp increase to 227 (96-98% probability of HLH) [7]. High-dose intravenous methylprednisolone, targeted interleukin-1 (IL-1) receptor blockade with anakinra, and trimethoprim-sulfamethoxazole prophylaxis were immediately initiated in accordance with current recommendations for severe secondary HLH and immune-related adverse events [5,8-10].

Despite aggressive medical management, her renal failure progressed to Stage 3 AKI with a peak serum creatinine of 560 µmol/L, necessitating continuous renal replacement therapy. A bone marrow biopsy performed to evaluate persistent multilineage cytopenias demonstrated chemotherapy-induced dyspoiesis without morphological evidence of tissue haemophagocytosis. A specialist multidisciplinary team (MDT) consensus concluded that the absence of bone marrow haemophagocytosis did not exclude secondary HLH, given the overall clinical and biochemical picture, including marked hyperferritinaemia, hypertriglyceridaemia, cytopenias, transaminitis, and an HScore of 227 (96-98% probability of HLH). Targeted immunosuppression was therefore continued [5,8]. The complex dynamic interplay between her hyperinflammatory markers, cytopenias, and declining renal function throughout this period is illustrated in Figure 1.

**Figure 1:**
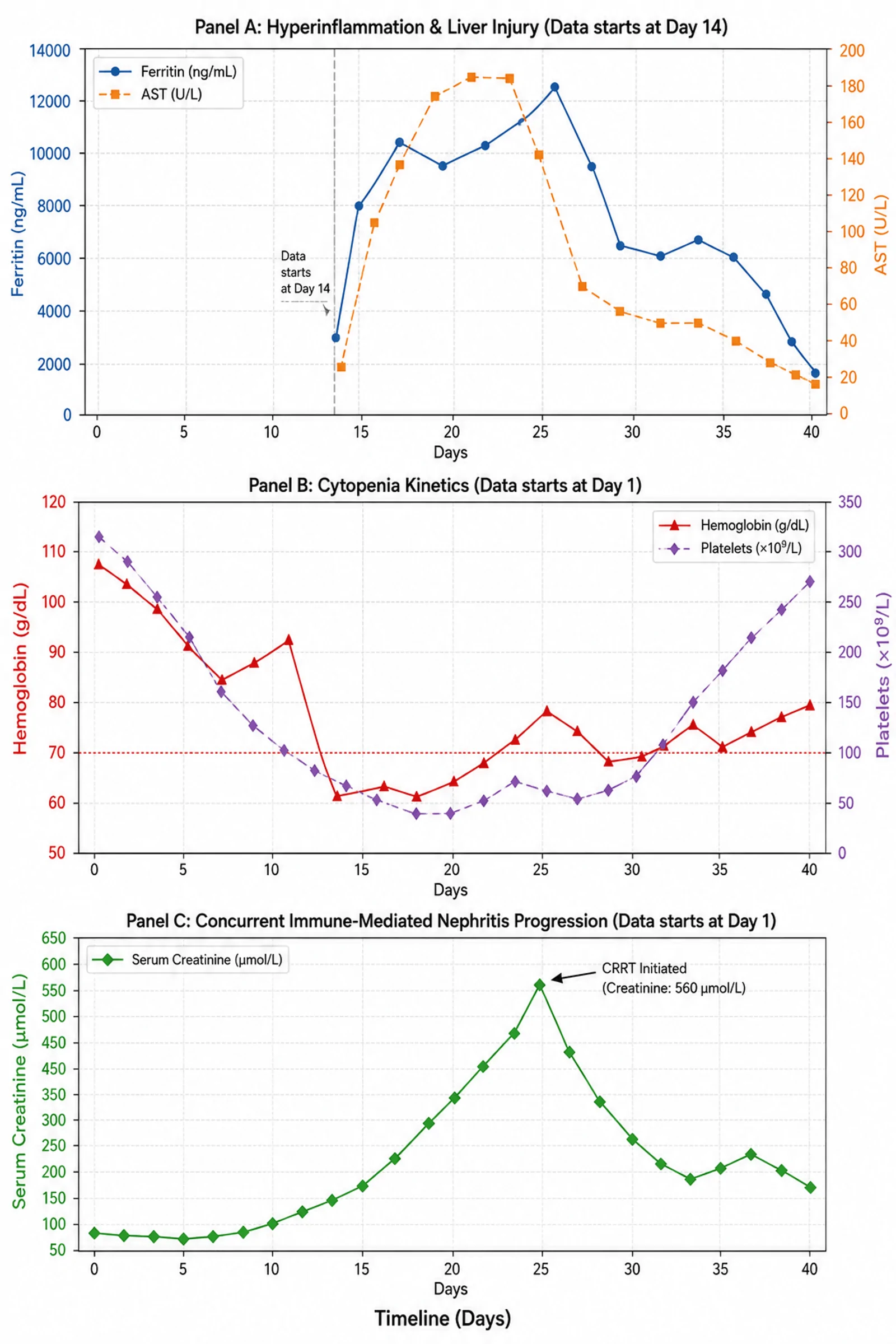
Comparative chronological profiling of inflammatory (ferritin, AST), hematologic (Hb, platelets), and renal (creatinine) biomarkers. Panel A: hyperinflammation and hepatic injury dynamics represented by serum ferritin (blue line, ng/mL) and aspartate aminotransferase (AST; dashed orange line, U/L). Panel B: Cytopenia kinetics demonstrating progression of anemia (red line, hemoglobin g/L) and thrombocytopenia (dashed purple line, platelets × 10^9/L). Panel C: Course of concurrent immune-mediated nephritis showing serum creatinine elevation (umol/L), peak severity necessitating CRRT initiation at 560 umol/L, and subsequent renal recovery.

Following the addition of targeted IL-1 inhibition and pulse corticosteroids, the patient demonstrated rapid clinical improvement. Inflammatory markers trended downward, renal parameters stabilised, and continuous renal replacement therapy (CRRT) was successfully discontinued. The working diagnosis was confirmed as pembrolizumab-induced secondary HLH with overlapping immune-mediated nephritis [6,9,10].

To manage her immune-mediated nephritis and serve as a steroid-sparing agent, oral mycophenolate mofetil (MMF) was introduced at 500 mg twice daily and uptitrated to 1.5 g twice daily, in keeping with contemporary guidelines for steroid-refractory immune checkpoint inhibitor nephritis [9,10]. Her urine PCR subsequently decreased to 529 mg/g. Follow-up laboratory evaluations demonstrated resolution of her hyperinflammatory state, with her HScore falling to 79 (<1% probability of HLH) [7] and her serum creatinine recovering to 134 µmol/L. Anakinra was discontinued, intravenous methylprednisolone was transitioned to oral prednisolone at 130 mg daily, and she was safely discharged on an oral corticosteroid taper alongside maintenance MMF therapy. Following recovery from the acute hyperinflammatory syndrome and AKI, the patient remained under close follow-up with the oncology team, with regular early blood tests to monitor her clinical recovery and response to immunosuppressive therapy. She remains suitable for definitive breast cancer surgery once clinically stabilised and her immunosuppressive treatment has been sufficiently tapered, with surgery anticipated in approximately three to four weeks. She will be reviewed in the breast surgical outpatient clinic for operative planning. Close ongoing oncology follow-up will continue as part of her longer-term breast cancer management and surveillance.

## Discussion

Immune checkpoint inhibitor-induced hemophagocytic lymphohistiocytosis (ICI-HLH) is a rare, life-threatening hyperinflammatory emergency driven by unbridled T-cell activation and systemic cytokine release [5,11]. Systematic reviews of ICI-induced HLH demonstrate that it is a distinct, drug-related entity requiring heightened clinical vigilance [5]. Furthermore, global pharmacovigilance databases report that HLH occurs significantly more frequently with ICIs than with other oncology drug classes [5]. However, because its clinical presentation closely mirrors that of neutropenic fever, severe sepsis, and line sepsis, clinicians frequently default to expanding broad-spectrum antimicrobial coverage. As illustrated in this case, persistent fevers despite documented microbiological clearance serve as a critical diagnostic turning point, prompting a shift away from a refractory infectious etiology toward a primary hyperinflammatory driver.

Distinguishing adult secondary HLH from overwhelming sepsis presents a major diagnostic challenge. While the traditional HLH-2004 criteria are widely recognized, they rely heavily on specialized assays-such as soluble CD25 (sCD25) levels and natural killer (NK) cell activity-that often suffer from prolonged turnaround times in acute settings [5,12]. Conversely, validated diagnostic tools like the HScore offer a rapid, weighted probabilistic assessment utilizing routinely available laboratory parameters [7]. Serial HScore calculations captured a dynamic rise from 149 to 227 in this patient, providing the objective biochemical justification required to move past a conservative "watch-and-wait" approach and initiate targeted immunosuppression.

Notably, the patient exhibited neither splenomegaly nor bone marrow hemophagocytosis. Large systematic reviews and pharmacovigilance studies confirm that classic diagnostic features are often absent in drug-induced hyperinflammation; for instance, splenomegaly is reported in less than one-third of ICI-HLH cases [5]. Because tissue hemophagocytosis is frequently patchy, operator-dependent, and delayed in appearance, its absence does not exclude the diagnosis [5,12]. Systematic reviews of drug-induced HLH (Rx-HLH) secondary to ICIs demonstrate that atypical presentations are common, frequently lacking classic features such as organomegaly or histopathological confirmation [5,12]. Clinicians must therefore prioritize overall systemic inflammatory trends-such as rapidly escalating hyperferritinemia-over waiting for definitive tissue biopsy findings to avoid treatment delays. Furthermore, while Epstein-Barr virus (EBV) is a recognized trigger for secondary HLH, the low-level viremia (1,330 copies/mL) observed here was clinically interpreted as transient reactivation in an immunocompromised host rather than the primary driver, which was mediated by pembrolizumab-induced T-cell disinhibition.

Managing ICI-HLH in the setting of concurrent organ failure requires a tailored approach. Standard HLH-94 protocols rely heavily on etoposide, a potent cytotoxic agent that carries significant myelosuppressive and nephrotoxic risks [5,12]. Given the patient’s concurrent Stage 3 AKI requiring CRRT due to immune-mediated nephritis, an organ-preserving regimen was critical. Interleukin-1 (IL-1) blockade with anakinra has emerged as a targeted biological alternative to cytotoxic therapy, directly interrupting the upstream IL-1-mediated cytokine storm without compounding cytopenias or worsening renal dysfunction [11].

Following initial stabilization, mycophenolate mofetil (MMF) was added as a steroid-sparing agent with dual therapeutic utility, addressing both the underlying hyperinflammation and the pembrolizumab-induced nephritis. While high-dose systemic corticosteroids remain the cornerstone of frontline management for ICI-related organ toxicities [10,13], MMF serves as a preferred second-line immunomodulatory agent for steroid-refractory cases or overlapping immune-mediated toxicities where other agents like infliximab are contraindicated due to multiorgan failure or sepsis risks [10,13]. This tailored immunomodulatory combination facilitated rapid clinical recovery, complete liberation from CRRT, and improvement of her creatinine to 134 µmol/L, allowing for the discontinuation of anakinra and safe discharge on oral prednisolone (130 mg).

## Conclusions

This case highlights the diagnostic challenge of differentiating ICI-associated HLH from persistent infection in patients undergoing neoadjuvant chemotherapy. Serial HScore monitoring may support early recognition of evolving hyperinflammation. In this patient, the temporal association with pembrolizumab exposure, exclusion of alternative infectious triggers, and concurrent immune-mediated toxicity supported a probable association with pembrolizumab-associated HLH. When managing overlapping toxicities such as HLH and presumed immune-mediated nephritis, targeted biological therapy with anakinra and organ-preserving immunosuppression with MMF were associated with resolution of hyperinflammation and subsequent renal recovery. Multidisciplinary input was critical in guiding safe and effective management of the overlapping immune-related toxicities and severe organ dysfunction.
